# Human dexterity and brains evolved hand in hand

**DOI:** 10.1038/s42003-025-08686-5

**Published:** 2025-08-26

**Authors:** Joanna Baker, Robert A. Barton, Chris Venditti

**Affiliations:** 1 Division of Ecology and Evolutionary Biology, University of Reading, Reading, UK; 2 School of Life, Health and Chemical Sciences, The Open University, Milton Keynes, UK; 3 Department of Anthropology, Durham University, Durham, UK

**Keywords:** Evolution, Phylogenetics

## Abstract

Large brains and dexterous hands are considered pivotal in human evolution, together making possible technology, culture and colonisation of diverse environments. Despite suggestions that hands and brains coevolved, evidence remains circumstantial. Here, we reveal a significant relationship between relatively longer thumbs – a key feature of precision grasping - and larger brains across 95 fossil and extant primates using Bayesian phylogenetic methods. Most hominins, including *Homo sapiens*, have uniquely long thumbs, yet they and other tool-using primates conform to the broader primate relationship with brain size. Within the brain, we surprisingly find no link with cerebellum size, but a strong relationship with neocortex size, perhaps reflecting the role of motor and parietal cortices in sensorimotor skills associated with fine manipulation. Our results emphasise the role of manipulative abilities in brain evolution and reveal how neural and bodily adaptations are interconnected in primate evolution.

## Abstract (web summary)

Thumbs and brains coevolved in primates. Across living and extinct species, longer thumbs predict bigger brains, highlighting the neural cost of dexterity.

## Introduction

Manual dexterity has long been celebrated as a cornerstone of our own evolutionary success, facilitating technological innovation, cumulative culture, and rapid cultural adaptation to variable environments e.g., see refs. ^1–4^. While numerous suggestions have been made about the potential link between manual dexterity and cognition^1,5^, the ways in which natural selection acted to shape the human hand and its coevolution with the brain remain poorly understood. Here we set out to examine how the coevolution of complex manipulative behaviours and brain size^1^ in primates is reflected in morphology – the traits that change in response to natural selection in order to facilitate such behaviour. Using a Bayesian phylogenetic comparative approach to studying behaviour-correlated morphological features, we can directly test for relationships in extinct species like our own ancestors – in which the behaviours and brain mechanisms themselves are unobservable.

**Fig. 1:**
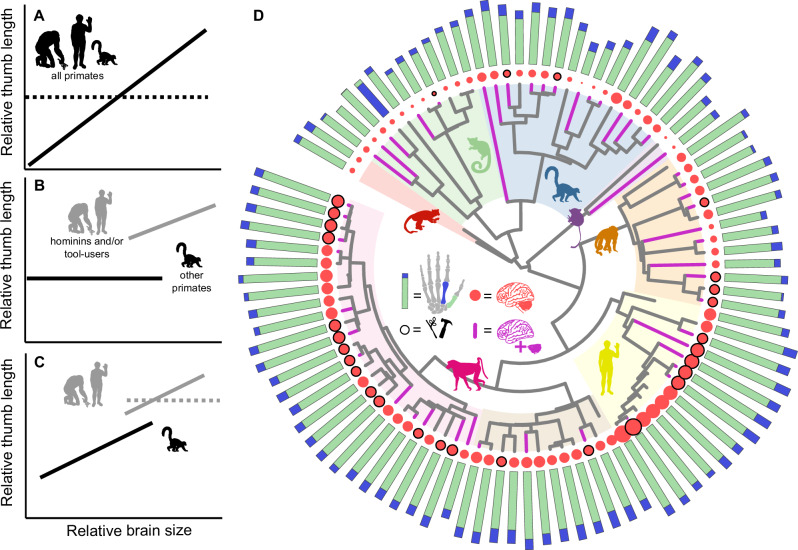
Data and potential scenarios for the coevolutionary relationship between thumb length and brain size across primates. **A** In this (expected) scenario, relative brain size and thumb length co-evolved across all primates (solid line). If these two traits are unlinked, we would observe no relationship (dashed line). **B** Alternatively, we may see a scenario in which a relationship exists only for hominins and/or tool-users. **C** Finally, it is possible that brain size and thumb length coevolved across all primates, but there is a shift in the intercept of the relationship between hominins and/or other tool-using species, which might be the case if there was some reorganization of the neuro-behavioural basis of manipulation. **D** Phylogenetic tree of the 95 species used in the main analyses. Manual dexterity is measured using the relationship between the length of the first metacarpal (MC1, green) and the second metacarpal (MC2, blue) – the length of both bones is shown by the bars at the tips of the tree (shorter bone superimposed on top). Whole brain size is represented by red circles at the tips of the tree, with species with documented tool-use outlined in black. Species for which we have both cerebellum and neocortex volumes are indicated by purple branches. Silhouettes represent major primate clades and are for illustrative purposes only: Adapiformes (*n = *2, red); Lorisiformes (*n = *11, green); Lemuriformes (*n = *16, blue); Tarsiiformes (*n = *2, purple); Platyrrhini (*n = *13, orange); apes (*n = *13, yellow); Colobinae (*n = *12, brown); Cercopithecinae (*n = *26, pink).

Beyond hominins, tool use is observed in many species^6–13^, and is in fact but one manifestation of skills related to extractive foraging^14,15^ – which are even more widespread. Previous work has found that manipulation behaviors co-evolved with brain size in primates^1,16^ Here we ask how this may be related to variation in hand morphology, which allows us also to examine fossil species in a phylogenetic context. A variety of anatomical factors^17,18^ affect manipulative ability including thumb robusticity^4^ and relative thumb length^18,19^, as well as more complex aspects of hand proportions^20^. Here, we focus on relative thumb length. Whilst we recognise the additional role of other features of the hand, an increased ability to manipulate small objects is enhanced by long thumbs^19,21,22^ – particularly relative to the index finger^23^. Longer relative thumbs facilitate greater opposability^24^ – and *Homo sapiens* is noted to have both longer thumbs compared with other apes^25,26^ as well as enhanced manipulative ability^2,17,24^. However, while *H. sapiens* possesses a uniquely refined precision grasp^24,27^, there are varying degrees of opposability across primates^23^ and precision grasping behaviours are found within other species with only pseudo-opposability such as capuchins^21,26^. If fine manipulative abilities require enhanced sensorimotor control with an associated neural processing cost, then we would expect to see a general co-evolutionary relationship between thumb length and brain size across the primate order (Fig. 1A). Indeed, this may explain some of the marked variation in relative brain size among primates and the trend for this to increase through time^28^.

However, if long thumbs are a hominin-specific adaptation associated with refined precision grasping^18,21,27^ and/or the advent of tool culture^17,18,29^, then we would expect to observe a relationship only amongst hominins – along with an increase in thumb length (Fig. 1B). A third possibility is that long thumbs were advantageous for tool use in other clades too, predicting an increase in thumb length in hominins associated with habitual tool use and in other tool-using primates (Fig. 1B). In this case, a primary link between brain size and dexterity is driven by sensorimotor specialisation specifically for tool use. Finally, if increased thumb length in tool users or hominins arose alongside a more general relationship with manipulative abilities and brain size across all other primates (Fig. 1C), this implies a need for longer thumbs that requires no additional neural processing. In this scenario, this implies that alternative factors may have driven the evolution of both traits – or that some element of overall brain size has been otherwise reduced.

Here, we conduct the first empirical test of the hypothesis that brain size and hand morphology were linked during primate evolution, using comparative phylogenetic analysis and a dataset of 95 fossil and contemporary primate species spanning all primate diversity (Fig. 1D, Supplementary Data 1). We test the hypothesis that selection for sensorimotor control of manually dextrous behaviours modified the thumbs of tool-using primates and had an associated neural cost reflected in whole brain size. We additionally test whether thumb length may have differentially evolved with respect to brain size amongst *H. sapiens* and our extinct ancestors using phylogenetic outlier tests^30^.

## Results

**Fig. 2:**
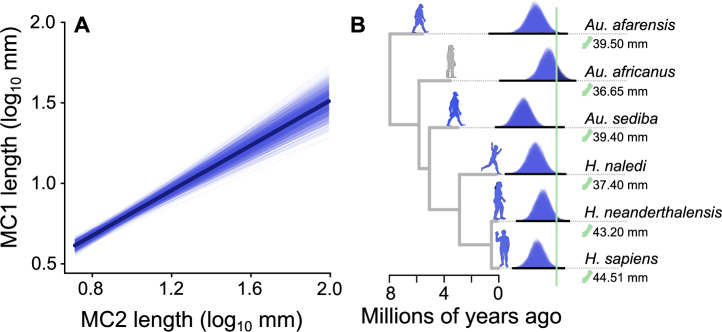
The relationship between finger length and thumb length across primates. **A** A random sample (*n* = 25) of fitted slopes from our finger-only model (MC1 ~ MC2) are plotted across a random sample of *n* = 50 trees. The median fitted relationship is superimposed. **B** The hominin phylogeny (using a single representative from the sample) is plotted along with the posterior distributions of imputed thumb lengths from the finger-only model. There are 100 distributions for each hominin for each model – one for each of the topologies in the sample. Outliers are identified when the posterior distribution of estimated thumb lengths overlaps the true value by less than 5%. The real thumb length of each species is indicated by the green line. Silhouettes are shown for representative purposes only and are not to scale. Silhouettes are shown for representative purposes only and are not to scale but are coloured according to whether they are identified as an outlier (grey = non-outlier, blue = outlier).

Results from our Bayesian phylogenetic generalized least squares (PGLS) regression models implemented in BayesTraits^31^ and accounting for phylogenetic uncertainty by using a sample of dated trees (see Methods) support the expectation that thumb length and finger length are strongly linked across all primates (Fig. 2A, *n* = 95, *finger-only models*). The relationship is significant in 100% of our tree sample (see Methods), with a median slope parameter ranging between 0.87 and 0.89 across the sample. There is high phylogenetic signal, with a median λ of 0.81–0.89. Full parameter ranges are reported in Supplementary Table 1. Results are qualitatively identical (i.e., we draw the same fundamental conclusions based on statistical significance) using alternative bones and digits (Supplementary Note 1, Supplementary Tables 2–7).

Using a phylogenetic imputation procedure^30^, we then identified which hominins had longer thumbs than expected given intrinsic hand proportions across primates. Given the finger-only model estimated across all non-hominin primates, we find that all but one hominin species (*Australopithecus africanus*) are significant outliers compared to non-hominin primates (Fig. 2B, see methods). As expected^26^, hominin thumbs are significantly longer than those of other primates. The general primate-wide relationship predicts hominin species to have much shorter thumbs than are actually observed.

**Fig. 3:**
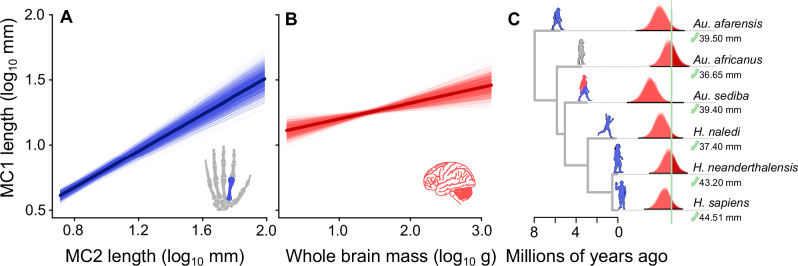
The relationship between thumb length, finger length, and brain size across primates. In panels (**A,B**), a random sample (*n* = 25) of predicted relationships from our whole-brain model (MC1 ~ MC2 + whole-brain) are plotted across a sample of *n* = 50 random trees, holding the unplotted variable at its mean value. The median predicted relationships calculated across all trees are superimposed. The predicted relationship between thumb length and finger length is shown in (**A**) and the predicted relationship between thumb length and whole brain mass is shown in (**B**). **C** The hominin phylogeny (using a single representative from the sample) is plotted along with the posterior distributions of imputed thumb lengths from the whole-brain model. There are 100 distributions for each hominin for each model – one for each of the topologies in the sample. Outliers are identified when the posterior distribution of estimated thumb lengths overlaps the true value by less than 5%. The real thumb length of each species is indicated by the green line. Silhouettes are shown for representative purposes only and are not to scale but are coloured according to the models in which they are identified as an outlier (grey = none, blue = finger-only, red = whole-brain).

We then conducted an additional set of PGLS models to test whether variation in thumb length is associated with brain size after accounting for allometry (using intrinsic hand proportions i.e. finger length). In these *whole-brain models* (*n* = 95), we find that the relationship between thumb length and finger length is maintained, with similar significance to that found in our finger-only models (median β_[finger]_ = 0.69–0.72, p_x_ < 0.05 in 100% of trees, Fig. 3A). This model also retrieves a significant positive association between thumb length and brain size (median β_[brain]_ = 0.11-0.13, p_x_ < 0.05 in 100% of trees, Fig. 3B). There is high phylogenetic signal in this model (median λ = 0.78–0.87) and the results are qualitatively identical excluding all hominins (*n* = 6). We find that there is still a significantly positive relationship between relative thumb length and brain size (median β_[brain]_ = 0.08–0.11, p_x_ < 0.05 in 100% of trees) as well as thumb length and finger length (median β_[finger]_ = 0.74–0.78, p_x_ < 0.05 in 100% of trees) across all non-hominin primates. That is, hominins are not driving the observed association between thumb length and brain size (Supplementary Table 1).

We then repeated the phylogenetic imputation procedure using our whole-brain model estimated across all non-hominin primates. After accounting for brain size, no hominin species (except *Australopithecus sediba*) is identified as an outlier to the thumb length and brain size relationship across all other non-hominin primates (Fig. 3). In all but *A. sediba*, the posterior distribution of estimated thumb lengths overlaps the true value by more than 5%, in more than 95% of trees.

The hypothesis that longer thumbs are specifically advantageous with regards to tool use predicts a difference in thumb length between tool-using primates and those never observed to use tools. To test this prediction, we assessed the relationship between thumb length and tool use - defined as the non-social use of external objects to alter the properties of a target object or medium^32,33^. Using a comprehensive compilation of observed tool-use across the animal kingdom^32^ to identify tool-using primates, we ran an additional PGLS analysis that included thumb length as a response variable and finger length, brain size and tool use as covariates. In these *tool-use models*, we find no significant difference in the slope of the relationship between thumb length and brain size for *n* = 28 extant primates that have been observed to use tools (p_x_ > 0.05 for both the intercept and slope difference in 99% of trees). Additionally, there is no mean difference in the thumb lengths of tool-using species either before or after accounting for brain size (see Supplementary Note 2). The inclusion of *H. sapiens* makes no qualitative difference to the results, and the overall relationships are qualitatively identical to those in our whole-brain models.

We additionally tested two alternative ways of defining tool use, all obtained from the same source^32^. We find the same results for species who exhibit “true tool use”, where objects are explicitly manipulated out of their original context^33,34^ as well as for species observed to explicitly manufacture or modify objects prior to use^32,35^. Finally, we ran two additional versions of each tool-use model excluding (i) species where either only a single individual has been observed using or making tools, or (ii) where observations came only from captive animals^32^. All alternative definitions and exclusions resulted in qualitatively identical conclusions.

Note that all the variables we include in our models are significantly associated with body size and thus we did not include body size as a covariate to avoid issues with multicollinearity. All analyses still account for size in the form of intrinsic hand proportions – by including the length of the second digit (MC2). However, when we tested models that additionally incorporated body size, we found it to be non-significant in 98% of topologies. As our results remain qualitatively identical when body size is included, here we present our results without body size.

The above results are all presented where finger length is represented by the second digit – which is generally considered to be an important indicator of manual dexterity^23,36^. However, other papers have demonstrated and highlighted the importance of other digits in grasping abilities and dextrous behaviours^18^. From a clinical perspective, the first and third digits are often used to derive functional dexterity metrics in humans e.g., see ref. ^37^ wherein precision handling is generally referred to as manipulation using the thumb and second or third digits^38^. Both third and fourth metacarpals have also previously been used to explicitly study hand size and proportions amongst hominins and other apes e.g., see refs. ^26,39,40^. More recently, it has been demonstrated that the fifth digit is likely to have played a key role in precision grips associated with hominin tool use and production^41–43^. For this reason, we also conducted our finger-only and whole-brain models (both with and without hominins and body size) using the metacarpals of each of the other digits (MC3-MC5) as our measure of finger length – as well as the proximal phalanges of all digits where available. In these models (presented in full in Supplementary Tables 2–7), we reach the same qualitative conclusions as those made using the second metacarpal – that there is a significant and strong relationship between brain size and thumb length.

## Discussion

### Thumb length and dexterity

Our results imply a robust association between brain size and manual dexterity. One of the advantages of using anatomical data such as bone length as an indicator of behaviour is that it is subject to much less error in measurement than behavioural observations^44^. Furthermore, anatomical features are much more likely to be directly subject to selective pressure and thus are directly related to the neural processes that control them in order to produce adaptive behaviours. However, whilst having longer relative thumbs clearly represents a key component of enhanced manipulation ability^19,21,22^, it does not fully capture the complexity of primate variation in dextrous behaviour and ability. A range of morphological traits have been demonstrated to influence thumb dexterity beyond relative thumb length^4,18,20^, including (among others) relative proportions and morphology of other digits^20,41^, bone shape and structure^4,45,46^, and bone traits associated with soft tissues such as muscle attachment sites (entheses)^42,47,48^. Primate dexterity is clearly facilitated by more than just thumb length alone; whilst thumb length can provide us with some general insights, detailed musculoskeletal and biomechanical modelling studies can provide us with critical insights into the various other factors driving dexterity and mobility^4,23,43,49^.

The multifactorial nature of primate and, more specifically, hominin, dexterity may mean that having a long thumb or even high joint mobility in isolation is not sufficient for high manipulation capability from a biomechanical standpoint^23^. However, it does not mean that thumb length is not still linked to manual dexterity. In line with this, here we demonstrate that relative thumb length is strongly and significantly linked to measures of dexterity derived from biomechanical and kinematic models (Supplementary Note 3). In a sample of 41 primate species, we tested the relationship between *peak manipulation workspace* – a biomechanical measure of dexterity that defines the range of motion a small object can be freely moved between the thumb and index finger *-* and thumb length. We find that thumb length significantly predicts peak manipulation workspace (β_[workspace]_ = 0.57–0.61, p_x_ < 0.05 in 100% of topologies) – an association that is unaffected by the inclusion of *H. sapiens* or optimum object size.

In order to further strengthen our interpretation of our results as evidence for coevolution of brain size and manual dexterity (as indicated by thumb length), we then tested for an association between brain size and peak workspace running phylogenetic regression models in exactly the same way as we did for thumb length in our main analyses (replacing thumb length with peak workspace). In line with our main results, we find that brain size is a significant predictor of peak workspace (β_[brain]_ = 0.090, p_x_ < 0.05 in 100% of topologies). The result is qualitatively identical when *H. sapiens* are excluded (β_[brain]_ = 0.09–0.11, p_x_ < 0.05 in 100% of topologies).

Therefore, on the basis of both our thumb length and workspace analyses, we interpret our results to indicate sustained historical coevolution between brain size and dexterity across the primate order, reflecting significant neural costs of manipulation behaviours and helping to explain the rapid increases in brain size observed in hominins e.g., see ref. ^50^.

### Hominin dexterity in context

Hominins have much greater relative thumb length compared to other primates (Fig. 2) – and even other apes^25,26^. This has specifically been linked to refined precision grasping^18,21,27^ and used as an indicator for tool culture^18,29^ (reviewed in ref. ^17^), but the timing of the emergence of these behaviours and associated morphologies are highly contested^19,36,51–53^. We, however, find a primate-wide association between brain size and thumb length, indicating that thumb length is a more general measure of dexterity not specific to hominins. This is in line with suggestions that features of the hominin hand, including long thumbs, pre-date the origin of systematic tool production^26,36,51^. Our results provide no support for the idea that thumb lengths are sufficient morphological indicators of tool-use –either in hominins or across all primates. We therefore cannot make any inferences about tool-use in hominins from our results. However, our analysis does provide a framework in which future research may be able to identify outliers amongst hominin species (and others) in any measurable morphological feature involved in manual dexterity and – more importantly – how these have evolved in relation to brain size. For example, whilst thumb length in *Au. afarensis* has previously been debated in the context of precision grasping^54,55^, recent biomechanical analyses have revealed that this species was most likely unable to make stone tools based on the carpometocarpal joint of the fifth digit^43^. Therefore, testing a combination of simple morphological proxies (such as thumb length) along with metrics revealed in critical biomechanical modelling analyses^4,23,43,49^ reveal clear nuances in hominin hand evolution. What remains to be tested is how any of these link to brain size – which is becoming more plausible as data and model availability become increasingly more available.

Regardless, our results demonstrate coordinated change in both hands and brains and therefore confirm the prediction outlined in Fig. 1A. This is striking: whilst hominin thumbs are outliers amongst primates in terms of length (Fig. 2B), this is almost entirely explained by a general relationship across primates (Fig. 3). For example, although *A. africanus* does not have significantly long thumbs relative to finger length (Fig. 2B), it still conforms to the whole-brain relationship observed across all primates (Fig. 3C). That is, the combination of brain size and thumb lengths in this taxon leads to manipulative ability comparable to other hominins – as suggested by other studies^23,36^. In the absence of finger-length data for other hominins, we can only make robust statistical inferences for those species included in our dataset. However, it seems likely that most hominins will conform to the patterns observed in these species and across all other primates. For example, even though taxa like *H. floresiensis* have been noted to have particularly small brains^56,57^, they also conform to the patterns of brain size evolution observed across other species^50^. Regardless of this, whilst it is possible new data for other species may reveal individual outliers to the pattern, the overall evolutionary relationship between brain size and thumb length is unlikely to be impacted.

Notably, the only hominin that does not conform to the general relationship across primates is *A. sediba*, a species previously noted to have an unusually long thumb^23^. The thumb length of *A. sediba* remains an outlier amongst primates even after accounting for brain size (Fig. 3). Whilst at face value, such a long thumb would imply that *A. sediba* possessed greater dexterous abilities than other hominins, its deviation from the expected relationship with brain size reveals that this interpretation may not be so simple. Like *Au. sediba, H. naledi* has also been noted to have long thumbs e.g^58^. and relatively small brains falling within the range of *Australopithecus*^59^. However, we find no evidence that *H. naledi* is an outlier to the overall primate relationship. This likely reflects the differences in hand use between the two species – both in terms of manipulative ability and climbing strategy^60^. It is clear that *Au. sediba* possessed a repertoire of adaptations linked to both ape-like locomotion along with some form of dexterous manipulation^25,61–63^. Simply possessing a long thumb without additional neural processing costs is not likely to have supported some inherently spectacular manipulation ability. This is supported by biomechanical models revealing relatively inefficient thumb opposition in *A. sediba* compared to members of the genus *Homo*^4^ and estimated workspaces not exceeding the range of modern *H. sapiens*^23^. We speculate that the long thumbs of *Au. sediba* may therefore represent a combination of selective pressures on relative hand proportions – along with the possibility that different regions of the brain may have reduced in order to accommodate an increase neural processing associated with sensorimotor ability (and thus no overall increase in brain size).

### Brain size versus structural reorganization

The evolution of tool use has often been linked to morphologies associated both with improved dexterity^17,18,29^ alongside broader sensorimotor and cognitive changes^11,29^. However, there is also evidence to suggest that – alongside generalized patterns of brain size increase^50^ – functional brain reorganization was also important in primate and hominin evolution^64–68^. For example, reorganization in the frontal and/or parietal brain regions have been implicated in both dexterous behaviour^69^ and technological innovation^64^ amongst hominin species. Recent evidence has even demonstrated possible functional and anatomical overlap in brain activation patterns involved in both tool-use and language processing^70^.

Given the importance of brain reorganization and functional overlap between neural networks, it is possible, then, that we might observe more nuanced relationships between indicators of manual dexterity (such as thumb length) and neuroanatomy of individual brain functions or regions. Improvements in fine-grained visuo-motor processes such as visually guided manipulation are expected to be associated with expansion of brain regions mediating these processes. Substantial areas of the primate neocortex and cerebellum are involved in visuo-motor control, and coordinated expansion of these structures explains much of the variation in brain size among primates^71^. We would therefore expect these regions to be associated with the co-evolution of manual dexterity and brain size^71^.

**Fig. 4:**
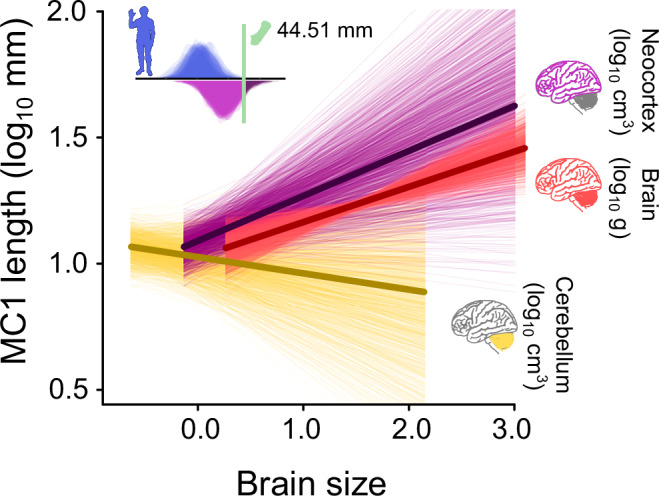
The relationship between brain region volume and thumb length. The relationships estimated from our brain-regions model (MC1 ~ MC2 + neocortex + cerebellum) using *n* = 49 extant primates. The fitted slopes are calculated holding the finger length, neocortex volume (for the cerebellum slopes), and cerebellum volume (for the neocortex slopes) at their mean values. Neocortex volume (purple) and whole-brain mass (red) are significantly positively associated with thumb length; the relationship between cerebellum volume and thumb length (yellow) does not differ from zero. Inset: The posterior distributions of imputed thumb lengths for the only extant hominin (H. sapiens) from our brain-regions model (purple) and an additional finger-only model limited to only the *n* = 49 species for which we have neocortex and cerebellum data (blue).

To test this idea, we used a reduced sample (*n* = 49, Fig. 1D) of primate species for which data were available, to determine the relationship between two brain regions (neocortex and cerebellum) and thumb length. In our *brain-regions model*s – in which we test the effects of both regions simultaneously – we find a significant positive relationship between thumb length and both finger length (median β_[finger]_ = 0.72–0.76, p_x_ < 0.05 in 100% of trees) and neocortex (Fig. 4, median β_[neocortex]_ = 0.16–0.20, p_x_ < 0.05 in 100% of trees). However, there is no such association found for the cerebellum (Fig. 4, β_[cerebellum]_, p_x_ < 0.05 in 0% of trees). The results are qualitatively identical when each brain region is considered in isolation (Supplementary Table 8).

We also find no link with an anatomical measure of binocular vision, convergence of the orbits (Supplementary Note 4). Our results are qualitatively identical without *H. sapiens* (Supplementary Table 1), and echoing our whole-brain model results, we find that modern *H. sapiens* are not outliers to the thumb-neocortex relationship in our brain-region models (Fig. 4, inset).

The lack of an association between cerebellum volume, binocularity, and thumb length is surprising, especially given the established role of the cerebellum and cortico-cerebellar networks in fine visuo-motor control and management of complex behavioural sequences^71,72^. Our brain-region relationships are more variable than those observed for the whole-brain (Fig. 4) and are potentially affected by smaller sample sizes. However, the conclusions are not affected by the exclusion of the apes, which exhibit rapid cerebellar expansion^73^, nor do the relationships diverge between haplorrhines and strepsirrhines despite differences in the sizes of their relative brain regions^74^. We therefore reveal the intriguing possibility that neural processes implicated in the evolution of manual dexterity across primates primarily involve neocortical regions^75^, such as frontal, motor and parietal cortices^76^. Although we did not predict this dissociation, a cortical contribution is in line with experimental evidence from modern *H. sapiens*, suggesting that motor cortex functioning and grey matter volume are both linked with manual dexterity and hand control^77–79^, and with fossil evidence for parietal cortex expansion in hominins^76^. Our finding that *H. sapiens* are not outliers (Fig. 4, inset) indicates that such observations may not be limited to our own species. An exciting avenue of future research would be to test this idea further as more data becomes available for other species.

Given the observed primate-wide relationship between neocortex and thumb length – and the fact that all extinct hominins would likely have been capable of some form of tool use (at least comparable to that observed in other primates), we would expect them to conform to similar patterns. We therefore refrain from drawing conclusions on our extinct relatives on the bases of our analyses. However recent years have begun to reveal the neural mechanisms of manual dexterity^80^ and tool-making behaviour^81^. These advances, combined with increasing availability of new modelling approaches^82^ and detailed data for fossil endocasts e.g^83^. may afford the opportunity to untangle exactly what neural mechanisms gave rise to modern dexterity. For example, studying markers for manual dexterity alongside brain regions undergoing structural reorganization in early *Homo*^83^ (including *H. habilis*^84^) may allow us to further understand whether a relationship exists for specific cerebellar regions connected to the motor cortex – and potentially distinguish between different types of dexterous behaviour.

### Concluding remarks

It is important to note that our analysis does not depend on the idea that brain size is a proxy for ‘general cognitive ability’, an idea that has been criticised^85^. Instead, we simply assume that variation in brain size – beyond that predicted by allometry – reflects selection on some aspect of neural processing, and that the sensorimotor control mechanisms associated with visually guided fine bimanual manipulation, and perhaps also action sequence planning, are expected to have neural processing costs^8,77,78,80,81^. The evolution of manipulative abilities may well have had far-reaching implications for cognition^14,15,70,86^ beyond the direct control of hand-movements per se. Such processes are likely to be reflected in neural processing costs and hence overall brain size. We are agnostic about what exactly those implications were and do not draw any strict distinction between sensorimotor control and cognition, regarding them as continuous with one another (see ref. ^87^). We also do not assume that all aspects of brain evolution involved simple changes in brain size, recognising that there are likely also to have been changes to neural mechanisms associated with manipulation that are not necessarily directly related to brain size, such as frontal and parietal cortex reorganization^64,83^.

As proponents of embodied cognition have suggested^86,87^, appreciating the links between bodily and brain adaptations is key to understanding neuro-cognitive evolution. Our results highlight the important role of the ability to manipulate food items or other objects in brain size evolution and emphasise the neocortical contribution to these behaviours. The results we present here go some way to explaining the uniquely hominin condition of having long thumbs. However, in isolation, no feature, including thumb length, should be considered as evidence for tool-use or tool-making behaviours^18^. A more complete picture might emerge with the increasing availability of more data for other, particularly earlier, hominins. Uncovering links between cognition and additional morphological features associated with dexterity e.g.^17,18^ may allow us to untangle the nuanced picture regarding the suites of traits associated with hominin tool use^18,23^ and their origins.

## Materials and Methods

### Primate phylogeny

All of our analyses are conducted in a phylogenetic context in order to account for the non-independence amongst species data points that can be attributed to shared evolutionary history^88^. All of our analyses are performed on a random sample of 100 of the most-parsimonious topologies obtained from the recently published comprehensive Euarchonta phylogeny including 894 fossil and extant primates^89^. As the original sample of trees is not time-calibrated, we dated these topologies using a tip-dating procedure adapted from the original paper^89^ and implemented in BEAST v2.7^90^. For full details on our tip-dating procedure, see the supplementary material^91^. As BEAST is implemented in a Bayesian framework, it gives a posterior distribution of dated trees for each of the 100 topologies in our sample. We created a single representative phylogeny for each topology by calculating a median tree based on the Kendall-Colijn distance metric^92^. We did this using the treespace library^93^ in R v.4.0^94^. All our analyses are performed on this sample of 100 median dated trees which are provided as supplementary data to this paper (Supplementary Data 2).

### Phenotypic data

We collected data on metacarpal measurements (lengths in millimetres) for primate species from the literature. We only included species found in the phylogeny^89^. We preferred compilation estimates (i.e., species-level data) but where specimen level data were included, we took a weighted average across all specimens (weighted, where possible, by number of specimens measured). Where individual specimens were measured by multiple sources, we preferred, arbitrarily, the most recently published source for each specimen. Our final dataset included finger bone measurements spanning 168 primate species, including 8 hominins (Figure S1). A full list of measurements and their sources can be found in the supporting information (Supplementary Data 1). Here, we use the length of the first metacarpal (MC1) and second metacarpal (MC2) as proxies for thumb and finger length respectively to measure relative thumb length and intrinsic hand proportions. However, our results remain qualitatively identical when proximal phalanges are used instead – or if we use any other digit instead of the 2^nd^ digit Supplementary Tables 2–7). Additionally, metacarpals are a robust and reliable indicator of overall digit length in our sample (Supplementary Note 1, Supplementary Figs. 2, 3) and are highly correlated with other bone measurements (Supplementary Fig. 4). We prefer to use bone length over recently proposed kinematic models^23^ for measuring manipulative ability as these are directly measurable quantities which are likely to face direct selection pressure from the environment, although our conclusions remain robust even when using these metrics (Supplementary Note 2). Whilst we do not have enough data to test associations for the distal or intermediate phalanges it is likely we would find similar associations.

We then collected brain mass data for these species. Brain masses or volumes were taken from the literature (see supporting information for full list of sources). In some cases – mostly for fossil taxa – we converted endocranial volumes to masses. Whilst endocranial volume is often converted to brain mass using the specific gravity of brain mass (1.036 g/mL)^95–97^, the majority of our extant data sample comes from a paper which uses a conversion of 1 g to 1 cm^3^ – and does not record which values were volume conversions^98^. For consistency, therefore, we use this conversion where necessary. The species for which this was done are recorded in the supporting information.

Tool-use data was taken from a published and comprehensive compilation^32^. Any species not included in this compilation were assumed to have not been observed using tools. This data is limited to only extant taxa. Whilst hominins would likely have been capable of tool-use to varying degrees, here we rely exclusively on observational data (testing hominins separately as described above). Our final dataset is graphically represented in Fig. 1. All continuously varying traits (brain size, metacarpal length, etc.) were logged before analysis.

### Statistics and reproducibility

Owing to the non-independence of species-level data attributable to shared ancestry^88^, we implemented all comparative analyses in a phylogenetic context. We used phylogenetic generalized least squares (PGLS) multivariate regression models implemented within a Bayesian Markov Chain Monte-Carlo (MCMC) framework to test for an association between manual dexterity and cognitive ability. Each analysis was conducted over a sample of 100 trees (see above). All models were run for a total of 1,000,000 iterations after convergence, sampling every 10,000 iterations. A wide uninformative prior was placed on all regression parameters (normal distribution with a mean of 0 and standard deviation of 5). We estimated the strength of phylogenetic signal using lambda^99^ in all models. All models were repeated multiple times (minimum *n* = 3) to ensure results were identical across replicates.

### Phylogenetic comparative analysis

Our first set of models (finger-only models, *n* = 95) tested the relationship between the lengths of MC1 and MC2. To assess the effect of brain size on relative thumb length, we included brain mass as an additional covariate – whilst also still including MC2 as a predictor to measure intrinsic relative thumb length and thus account for size. This model set is referred to as our whole-brain models (*n* = 95). We repeated both analyses excluding all hominins (*n* = 89, Supplementary Table 1). All analyses are conducted over all primates as a single group.

To identify which hominins (if any) were outliers in terms of their intrinsic hand proportions, we conducted a phylogenetic outlier test e.g., see ref. ^30^ using a phylogenetic imputation procedure^31^ to predict hominin thumb lengths. This predictive modelling approach simultaneously incorporates the parameters of a regression model as well as the phylogenetic position of each taxon. As with all our analyses, the imputations are calculated using PGLS regression models implemented within a Bayesian MCMC framework. We estimate the thumb length for each hominin given the parameters of each of our regression analyses (finger-only and whole-brain models) calculated across the rest of primates. We then assess whether each hominin species is an outlier using the full distribution of predicted values for thumb length– where the distribution overlaps the true value by less than 5% in more than 95% of trees, it can be considered a phylogenetic outlier.

To determine whether the relationship between brain size and thumb length is being driven by or affected by tool use, we ran an additional set of models (*tool-use models*) including the following predictors: MC2, a binary (dummy-coded) variable defining whether each species has been observed to use tools, and an interaction between the two variables. This explicitly tests for a different intercept and slope in the relationship between thumb length and brain size in species that have never been observed using tools compared to those which have. Note that we use three alternative ways of defining tool use, all obtained from the same source^32^. We additionally ran models excluding species where either only a single individual has been observed using or making tools, or where observations came only from captive animals^32^. All alternative definitions and exclusions resulted in identical conclusions (Supplementary Note 3).

To determine whether individual brain regions affected thumb length independently (brain-regions models, *n* = 49), we included both neocortex volume and cerebellum volume in a single model – along with MC2. Separating the effects of the neocortex and the cerebellum can be complicated owing to their strong correlation. We additionally repeated the model excluding *H. sapiens* (*n* = 48, Supplementary Table 1). Here, we included both regions in the same model here since together, the neocortex and cerebellum comprise a ‘unit’ responsible for the mediation of visuo-motor and sequential action control^100^. However, we find qualitatively similar results when each of the regions were studied in isolation: without *H. sapiens*, only the neocortex shows any significant association (Supplementary Table 8).

For all models, results are summarised across the sample of trees where the model is run separately for each tree. We assess significance of the parameters using two criteria: Firstly, the proportion of the posterior distribution that crosses zero (p_x_); where this proportion ≤ 0.05, we consider a variable to be significantly different from zero. Secondly, the first criterion must be met in at least 95% of topologies for us to consider a variable as significant. For comparison, we summarise parameter estimates using median values – and then to summarise across all trees, we report the range of observed medians_._

### Reporting summary

Further information on research design is available in the Nature Portfolio Reporting Summary linked to this article.

## Supplementary information

Supplementary Information

Description of Additional Supplementary Files

Supplementary Data 1

Supplementary Data 2

Reporting Summary

## Data Availability

All data are available in the main text or the supplementary materials, along with the sources from which they are obtained (Supplementary Data 1). All strepsirrhine hand and foot measurements were provided with permission to use in publication by Pierre Lemelin. Permission and access to this data can be obtained by contacting Pierre Lemelin directly.
