# Supplementary material for: Human dexterity and brains evolved hand in hand
Source: Commun Biol. 2025 Aug 26;8:1257. doi: 10.1038/s42003-025-08686-5 (PMC12381199; doi:10.1038/s42003-025-08686-5)
Supplement: Supplementary file 1 — Supplementary Information [file 42003_2025_8686_MOESM1_ESM.pdf]

Supplementary Information for:

Human dexterity and brains evolved hand in hand

Joanna Baker*, Robert A. Barton, Chris Venditti*

* Correspondence to: j.l.a.baker@reading.ac.uk and c.d.venditti@reading.ac.uk

Contents

Supplementary Methods 2

Data collection 2

Tip-dating the Euarchonta phylogeny 3

Supplementary Note 1: Using metacarpal length as a proxy for finger length 5

Supplementary Note 2: Tool use and thumb length 14

Supplementary Note 3: Peak workspace 14

Supplementary Note 4: Brain regions and binocularity 15

Supplementary References 17

Supplementary Methods

Data collection

Data was collected from the literature on the length of the proximal phalanges, intermediate phalanges, and metacarpals of digits 1-5 (**Supplementary Figure 1**). We recorded measurements for distal phalanges where they were noted in the sources from which we collected other measurements. Note that this dataset is non-exhaustive, as our search was targeted on the first and second metacarpals.

We started by collating all finger bone measurements from the literature (see **Supplementary Data 1** for all sources and measurements) – limited only to taxa found in the recently published Euarchonta phylogeny[1] (see **Supplementary Data 2)**. We recorded the lowest possible taxonomic designation for the record as provided by the original source as well as any specimen details. Where species names were not provided, but we had information on specimens, we looked up the specimen ID in the original collection for additional taxonomic information. For example, specimen ID UM_101963 is recorded as *Carpolestes* in the finger-length source[2], but the specimen record on MorphoSource records the full species name as *Carpolestes simpsoni*, associated with the original publication[3].

We then assessed duplicate measurements within individual sources, with the goal in mind to obtain a single specimen-level (or taxon-level) measurement for each bone from each source. Where there were measurements associated with both the left and right hand (e.g.[4]), we calculated a specimen-specific average. For datasets reporting measurements at the taxon level but for separate sides, we calculated an average in the same way. Additionally, for taxon-level datasets, multiple records from a single source were sometimes attributed to a single taxon in the phylogenetic tree (e.g. *Pan troglodytes troglodytes* and *Pan troglodytes schweinfurthii* are both considered as *Pan troglodytes*). For these, we calculated an average species value. Therefore, for each dataset, we obtained a single measurement for each bone for a given taxon or specimen.

However, in many cases, the same specimen or taxon has measurements reported across multiple sources. For individual specimens which had been reported across multiple sources, we obtained a single measurement for each bone by selecting a representative value from the range of available sources. To do this, we used the most recently published measurement for each specimen. This is with one exception: we preferred measurements from Kivell (2015)[5] over Prang (2021)[6] as specimen provenance was recorded in the former source in more detail. Where multiple estimates were published in the most recent year, we arbitrarily selected the first alphabetically. All datasets with restricted permissions[7] and personal communication (Rolian C., 2024 and Lemelin P., 2024) were only used if measurements were not available from an alternative source. For taxon-level datasets, it was not always clear which specimens were included in the calculation of the taxon value. Therefore, to avoid potential issues associated with pseudo-duplication (artificially giving more weight to an individual specimen), we selected a value from a single source for each taxon using the same procedure described above for specimens.

For our final analysis dataset, we preferred taxon-level data. If we already had data for our species at taxon-level, then we used the representative value obtained in the above step. If published taxon-level estimates were unavailable, we took an average of all available specimens. This resulted in a dataset with 178 individual unique taxa with length of at least one

of the finger bones (**Supplementary Data 1, Supplementary Figure 1**). We record in **Supplementary Data 1** whether the measurements were taken from the left or right side (or a mix) and from male or female individuals (or a mix) across bones.

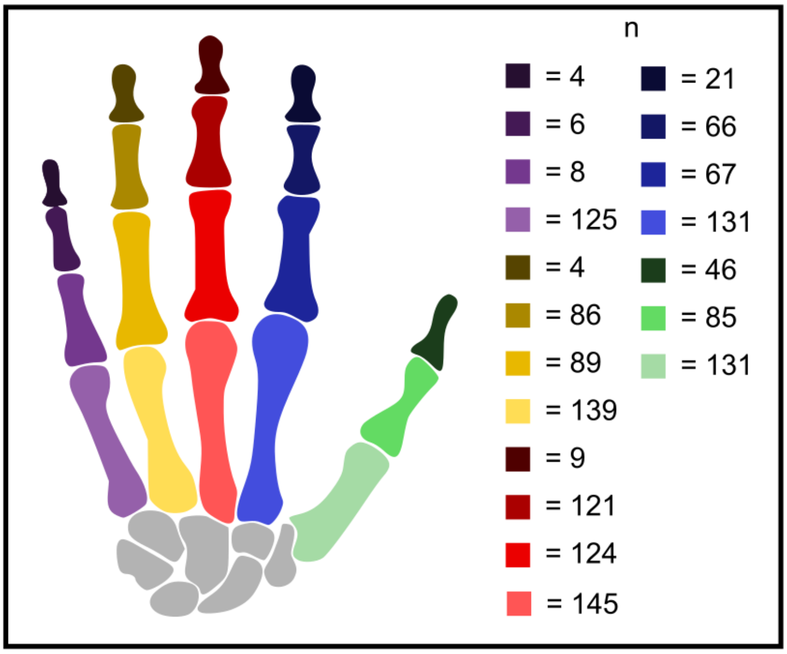

Supplementary Figure 1: A schematic representation of the finger bone length dataset used in this study. *Sample sizes are non-overlapping (i.e. not all species have all measurements).*

<u>Tip-dating the Euarchonta phylogeny</u>

All of our analyses are performed on a random sample of 100 of the most-parsimonious topologies obtained from the recently published comprehensive Euarchonta phylogeny including 894 fossil and extant primates[1]. As the original sample of trees is not time-calibrated, we dated these topologies using a tip-dating procedure adapted from the original paper[1] and implemented in BEAST v2.7[8]. This protocol was recently described in ref.[9].

Before dating, we removed several species owing to uncertain placement and/or taxonomic affiliation (Cercopithecini sp. indet. AUH 1321, Colobinae indet. KNM-BN 1251, Colobinae indet. KNM-TH 48368, *Cheracebus purinus*, *Tupaia* sp. UNSM 87244, and Dermoptera indet. Pkg 240 and Pkg 335). Our preliminary analyses demonstrated that inclusion of taxa with broad date ranges and uncertain placement resulted highly variable node ages depending on which clade the taxa fell within. We chose to exclude these species and therefore removed the influence of such taxa on our divergence dating analyses.

We then estimated branch length date variation by fixing the topology. We conditioned the fossilized birth-death process[10] on the root, and placed a uniform root calibration prior of between 66 and 130 million years as described in Dos Reis et al[11]. We used an optimized relaxed clock with the mean clock rate restricted to vary between 0.01 and 0.02 (just outside the ranges observed in the maximum credibility tree dated by Wisniewski et al[1] to facilitate faster convergence (though results are identical if this parameter is allowed to freely vary). The σ parameter of the log-normal distribution of the clock rates is drawn from an exponential prior with a mean = 1. Transition/Transversion rates were drawn from a gamma prior (α= 0.2, β = 0.5)

and (α= 0.2, β = 0.25) respectively. Finally, a uniform prior ranging between 0 and 1 was placed on the turnover rate and for the sampling proportion, we used a beta distribution (α = 5, β=90).

We then created a single representative phylogeny for each topology by calculating a median tree based on the Kendall-Colijn distance metric[12]. We did this using the treespace library[13] in R v.4.0[14]. We also, for our supplementary analyses (see below) and visualisation purposes, created a single overall reference tree by calculating an additional median tree based on our sample of trees. The sample of trees that we use for all our analyses is provided as **Supplementary Data 2** and is visualized in **Supplementary Figure 2** limited to the species for which we conduct our main analyses.

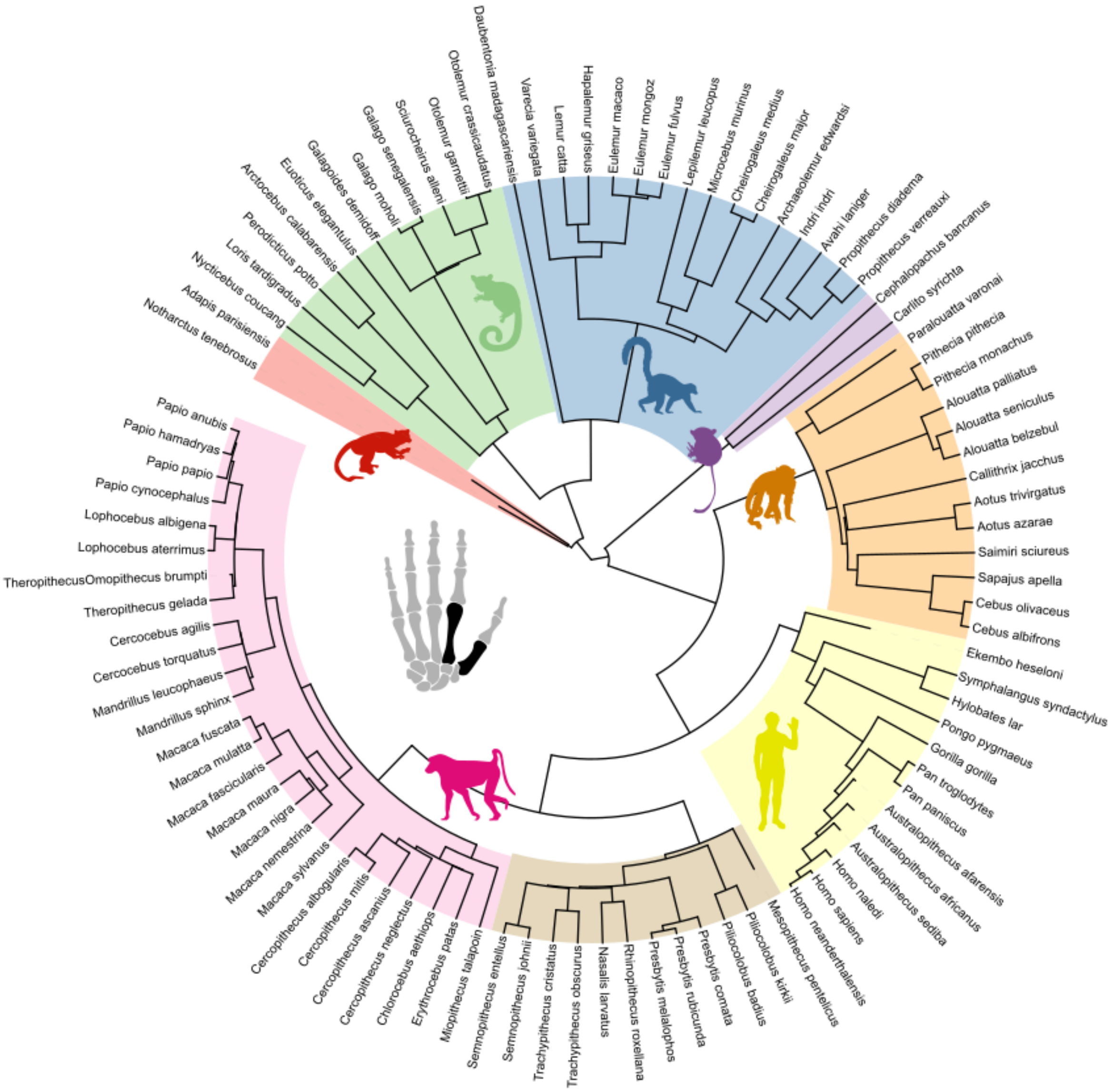

Supplementary Figure 2: Phylogenetic tree used in our main analyses. *Silhouettes represent major primate clades and are for illustrative purposes only: Adapiformes (n = 2, red); Lorisiformes (n = 11, green); Lemuriformes (n = 16, blue); Tarsiiformes (n = 2, purple); Platyrrhini (n = 13, orange); apes (n = 13, yellow); Colobinae (n = 12, brown); Cercopithecinae (n = 26, pink).*

Supplementary Note 1: Using metacarpal length as a proxy for finger length

One of the features associated with pad-to-pad precision grasping is a long thumb relative to the lengths of other fingers[15]. The ratio of thumb to index finger is often used as a proxy for manipulation ability and dexterity[16,17]. Here we measure both thumb and finger length using the metacarpals, significant predictors of adult and foetal body size[18,19] often used as a proxy for finger and thumb lengths across primates[20-22] – including in studies of dexterity[23]. Additionally, metacarpal length is strongly and significantly associated with digit length for all fingers (**Supplementary Figure 3**, all parameters highly significant). We do not conduct this analysis for the 5th digit owing to the paucity of available phalanx data.

We calculated digit length as the sum of the lengths of the proximal and intermediate phalanges (excluding metacarpal for comparison and the distal phalanx owing to availability). We then test the relationship between $\log_{10}$ digit length and $\log_{10}$ metacarpal length, using maximum-likelihood phylogenetic generalized least squares models implemented in caper[24], using a single representative phylogeny.

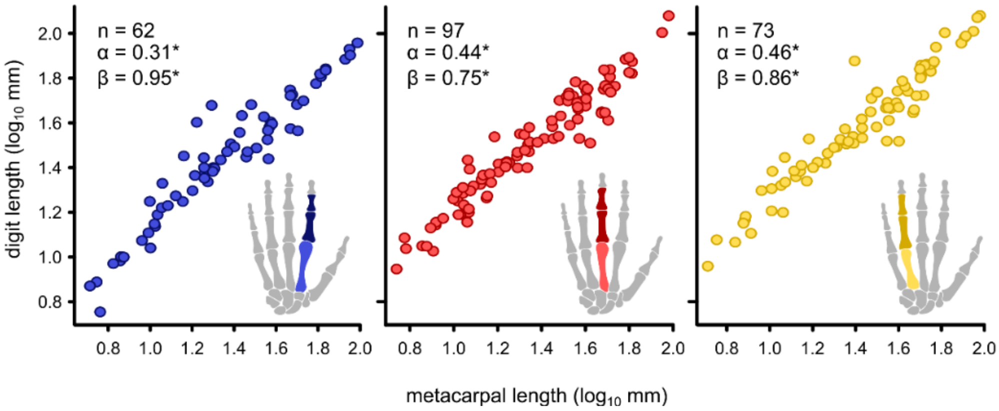

Supplementary Figure 3: The relationship between metacarpal length and digit length (measured as the sum of proximal phalanx and intermediate phalanx length) for digits 2-4. *Sample sizes, parameters and significance are indicated. *All parameters significant to $p < 0.0001$.*

We also find a significant relationship between the metacarpal and proximal phalanx of the thumb (**Supplementary Figure 4**). Notable outliers are colobus monkeys, which (along with spider monkeys) are excluded from our analyses owing to their rudimentary thumbs[25]. However, it should be noted that all conclusions we derive in the main text are unaffected by their inclusion.

Additionally, there is a strong and significant correlation between all bones in our sample (digits 2-4, **Supplementary Figure 5**). Again, we do not conduct this analysis for the 5th digit owing to the paucity of available phalanx data.

Finally, our results are qualitatively identical when phalanges are used instead of metacarpals – or if we use any other digit to test the relationship between brain size and relative thumb length. The results of all of our main tests are shown in **Supplementary Tables 1-7**.

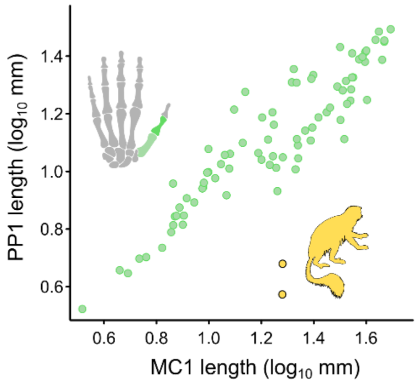

Supplementary Figure 4*: The relationship between the length of the metacarpal and the proximal phalanx in a sample of N = 175 primates with data for both bones.*

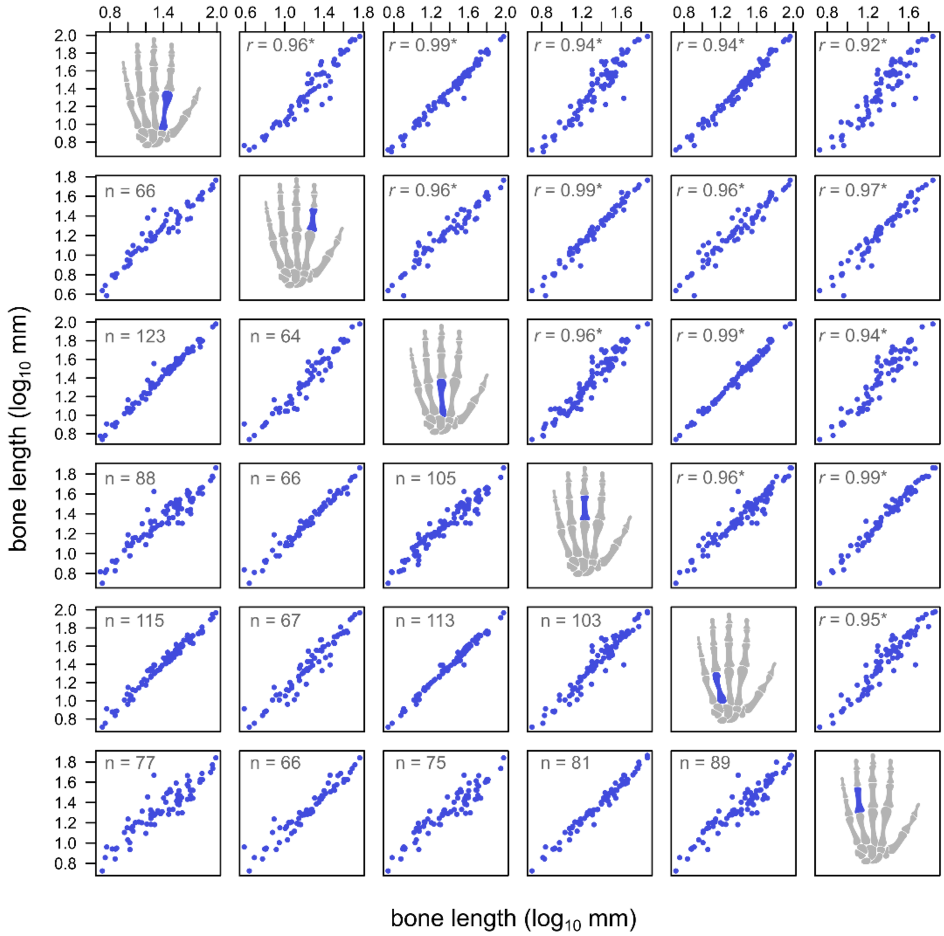

***Supplementary Figure 5**: **Pairwise correlation matrix between metacarpals and phalanges of the digits 2-4.** From top to bottom: MC2, PP2, MC3, PP3, MC4, PP4. Sample size for each comparison is shown in the lower half of the matrix and the correlation coefficient in the top half. All comparisons are highly significant, where * indicates p < 0.0001.*

Supplementary Table 1

Parameter estimates for results using MC1 as the proxy for thumb length and MC2 as the proxy for finger length. The brain-regions models include both neocortex and cerebellum as well as finger length as a covariate.

	R^2	λ	β	p_x	β	p_x	β	p_x
			Finger		Brain		Body	
1	0.82-0.84	0.81-0.89	0.87-0.89	0-0 [100]				
2	0.85-0.87	0.78-0.87	0.69-0.72	0-0 [100]	0.11-0.13	0-0 [100]		
3	0.85-0.87	0.81-0.9	0.64-0.69	0-0 [100]	0.08-0.1	0-0.03 [100]	0.04-0.05	0.03-0.12 [7]
4	0.85-0.87	0.85-0.91	0.9-0.91	0-0 [100]				
5	0.86-0.88	0.85-0.91	0.75-0.79	0-0 [100]	0.08-0.11	0-0.02 [100]		
6	0.86-0.88	0.86-0.92	0.71-0.76	0-0 [100]	0.06-0.08	0.05-0.12 [2]	0.02-0.04	0.09-0.22 [0]
7	0.91-0.92	0.92-0.97	0.72-0.76	0-0[100]	N: 0.16-0.20<br>C:-0.02-[-0.06]	N: 0-0.02 [100]<br>C: 0.18-0.33 [0]		
8	0.91-0.93	0.92-0.97	0.79-0.82	0-0[100]	N: 0.13-0.17<br>C:-0.03-[-0.07]	N: 0.05-0.08 [96]<br>C: 0.3-0.4 [0]		

Note. All values reported as the range of median values observed across the posterior distribution estimated for each of 100 trees. For p_x values, we also report the number of trees in which less than 5% of the posterior distribution crosses zero in square brackets. Models are as follows: 1, finger-only (N = 95); 2, whole-brain (N = 95); 3, whole-brain plus body (N = 95); 4-6 as in 1-3 but excluding hominins (N = 89); 7, brain-regions (N = 49); 8, brain-regions but excluding *H. sapiens*.

Supplementary Table 2.

Parameter estimates for results using MC1 as the proxy for thumb length and MC3 as the proxy for finger length.

	R^2	λ	β	p_x	β	p_x	β	p_x
			Finger		Brain		Body	
1	0.81-0.84	0.79-0.87	0.86-0.88	0-0 [100]				
2	0.85-0.87	0.73-0.85	0.66-0.68	0-0 [100]	0.13-0.14	0-0 [100]		
3	0.84-0.87	0.76-0.89	0.63-0.67	0-0 [100]	0.1-0.13	0-0.01 [100]	0.02-0.04	0.13-0.3 [0]
4	0.85-0.87	0.8-0.86	0.88-0.9	0-0 [100]				
5	0.86-0.88	0.79-0.87	0.71-0.74	0-0 [100]	0.1-0.12	0-0.01 [100]		
6	0.86-0.88	0.8-0.88	0.7-0.73	0-0 [100]	0.09-0.11	0.01-0.04 [100]	0-0.02	0.23-0.44 [0]

Note. All values reported as the range of median values observed across the posterior distribution estimated for each of 100 trees. For p_x values, we also report the number of trees in which less than 5% of the posterior distribution crosses zero in square brackets. Models are as follows: 1, finger-only (N = 92); 2, whole-brain (N = 92); 3, whole-brain plus body (N = 92); 4-6 as in 1-3 but excluding hominins (N = 85).

Supplementary Table 3

Parameter estimates for results using MC1 as the proxy for thumb length and MC4 as the proxy for finger length.

	R^2	λ	β	p_x	β	p_x	β	p_x
			Finger		Brain		Body	
1	0.77-0.81	0.88-0.98	0.83-0.86	0-0 [100]				
2	0.82-0.86	0.8-0.95	0.63-0.68	0-0 [100]	0.12-0.15	0-0 [100]		
3	0.82-0.86	0.8-0.96	0.63-0.68	0-0 [100]	0.11-0.15	0-0.01 [100]	0-0.02	0.2-0.49 [0]
4	0.85-0.87	0.86-0.95	0.89-0.91	0-0 [100]				
5	0.86-0.88	0.86-0.95	0.75-0.78	0-0 [100]	0.08-0.1	0-0.01 [100]		
6	0.86-0.88	0.86-0.96	0.75-0.79	0-0 [100]	0.08-0.1	0-0.04 [100]	-0.01-0.01	0.35-0.5 [0]

Note. All values reported as the range of median values observed across the posterior distribution estimated for each of 100 trees. For p_x values, we also report the number of trees in which less than 5% of the posterior distribution crosses zero in square brackets. Models are as follows: 1, finger-only (N = 107); 2, whole-brain (N = 107); 3, whole-brain plus body (N = 107); 4-6 as in 1-3 but excluding hominins (N = 100).

Supplementary Table 4

Parameter estimates for results using MC1 as the proxy for thumb length and MC5 as the proxy for finger length.

	R^2	λ	β	p_x	β	p_x	β	p_x
			Finger		Brain		Body	
1	0.81-0.85	0.84-0.98	0.8-0.83	0-0 [100]				
2	0.85-0.88	0.78-0.95	0.63-0.67	0-0 [100]	0.11-0.13	0-0 [100]		
3	0.84-0.88	0.79-0.97	0.61-0.66	0-0 [100]	0.08-0.13	0-0.02 [100]	0-0.03	0.12-0.47 [0]
4	0.87-0.9	0.79-0.95	0.83-0.86	0-0 [100]				
5	0.87-0.9	0.8-0.97	0.74-0.78	0-0 [100]	0.05-0.07	0.02-0.09 [53]		
6	0.87-0.9	0.8-0.98	0.73-0.78	0-0 [100]	0.04-0.07	0.07-0.21 [0]	-0.01-0.02	0.18-0.5 [0]

Note. All values reported as the range of median values observed across the posterior distribution estimated for each of 100 trees. For p_x values, we also report the number of trees in which less than 5% of the posterior distribution crosses zero in square brackets. Models are as follows: 1, finger-only (N = 103); 2, whole-brain (N = 103); 3, whole-brain plus body (N = 103); 4-6 as in 1-3 but excluding hominins (N = 96).

Supplementary Table 5.

Parameter estimates for results using PP1 as the proxy for thumb length and PP2 as the proxy for finger length.

	R^2	λ	β	p_x	β	p_x	β	p_x
			Finger		Brain		Body	
1	0.83-0.85	0.94-0.97	0.87-0.89	0-0 [100]				
2	0.86-0.88	0.91-0.95	0.7-0.74	0-0 [100]	0.11-0.13	0-0 [100]		
3	0.87-0.88	0.89-0.94	0.64-0.68	0-0 [100]	0.06-0.08	0.04-0.1 [5]	0.05-0.07	0.04-0.09 [24]
4	0.86-0.88	0.96-0.99	0.91-0.93	0-0 [100]				
5	0.88-0.89	0.94-0.98	0.76-0.8	0-0 [100]	0.09-0.11	0-0.02 [100]		
6	0.88-0.9	0.92-0.97	0.71-0.76	0-0 [100]	0.03-0.05	0.2-0.3 [0]	0.05-0.06	0.05-0.13 [1]

Note. All values reported as the range of median values observed across the posterior distribution estimated for each of 100 trees. For p_x values, we also report the number of trees in which less than 5% of the posterior distribution crosses zero in square brackets. Models are as follows: 1, finger-only (N = 60); 2, whole-brain (N = 60); 3, whole-brain plus body (N = 60); 4-6 as in 1-3 but excluding hominins (N = 55).

Supplementary Table 6

Parameter estimates for results using PP1 as the proxy for thumb length and PP3 as the proxy for finger length.

	R^2	λ	β	p_x	β	p_x	β	p_x
			Finger		Brain		Body	
1	0.8-0.82	0.78-0.89	0.84-0.86	0-0 [100]				
2	0.84-0.86	0.82-0.89	0.66-0.7	0-0 [100]	0.13-0.15	0-0 [100]		
3	0.84-0.86	0.8-0.87	0.61-0.65	0-0 [100]	0.08-0.1	0.01-0.05 [100]	0.04-0.05	0.07-0.15 [0]
4	0.86-0.87	0.71-0.8	0.87-0.89	0-0 [100]				
5	0.86-0.88	0.77-0.84	0.76-0.79	0-0 [100]	0.07-0.09	0.02-0.05 [99]		
6	0.87-0.88	0.71-0.8	0.7-0.74	0-0 [100]	-0.01-0.02	0.38-0.5 [0]	0.06-0.07	0.03-0.07 [63]

Note. All values reported as the range of median values observed across the posterior distribution estimated for each of 100 trees. For p_x values, we also report the number of trees in which less than 5% of the posterior distribution crosses zero in square brackets. Models are as follows: 1, finger-only (N = 72); 2, whole-brain (N = 72); 3, whole-brain plus body (N = 72); 4-6 as in 1-3 but excluding hominins (N = 66).

Supplementary Table 7

Parameter estimates for results using PP1 as the proxy for thumb length and PP4 as the proxy for finger length.

	R^2	λ	β	p_x	β	p_x	β	p_x
			Finger		Brain		Body	
1	0.81-0.84	0.96-0.98	0.87-0.9	0-0 [100]				
2	0.85-0.87	0.94-0.97	0.69-0.75	0-0 [100]	0.11-0.14	0-0 [100]		
3	0.85-0.87	0.93-0.97	0.66-0.72	0-0 [100]	0.08-0.12	0-0.02 [100]	0.02-0.04	0.09-0.2 [0]
4	0.85-0.87	0.96-0.98	0.91-0.94	0-0 [100]				
5	0.87-0.88	0.95-0.98	0.77-0.82	0-0 [100]	0.09-0.11	0-0.01 [100]		
6	0.87-0.88	0.94-0.97	0.74-0.79	0-0 [100]	0.05-0.08	0.04-0.13 [2]	0.02-0.04	0.1-0.23 [0]

Note. All values reported as the range of median values observed across the posterior distribution estimated for each of 100 trees. For p_x values, we also report the number of trees in which less than 5% of the posterior distribution crosses zero in square brackets. Models are as follows: 1, finger-only (N = 79); 2, whole-brain (N = 79); 3, whole-brain plus body (N = 79); 4-6 as in 1-3 but excluding hominins (N = 74).

Supplementary Note 2: Tool use and thumb length

If long thumbs are a hominin-specific adaptation associated with refined precision grasping [23,26,27] or the advent of tool culture[26,28,29], then we would expect to observe a relationship only amongst hominins – along with an increase in thumb length (**Figure 1B**). In a more general scenario, if long thumbs were advantageous for tool use – of any sort – then we would expect an increase in thumb length in hominins associated with habitual tool use as well as other tool-using primates but a reduced or no association with brain size amongst other primates (**Figure 1B**), wherein a primary link between cognition and dexterity is driven explicitly by sensory-motor specialization for tool use.

Species that use tools (n = 29) do not have significantly longer thumbs than other primates (n = 77) $p_{x\ [tool\text{-}use]}$ is non-significant in 100% of tested topologies). This relationship is unaffected when we exclude species who have only been observed using tools in captivity (n = 13) or species with only a single observation of tool use (n = 7). The result is identical if we use alternative definitions of tool use: firstly, in species observed to use 'true' tools (n = 28) – where objects are explicitly manipulated out of their original context [30,31]; and secondly, in species observed to explicitly manufacture or modify objects prior to use (n = 12) [32,33]. Accounting for brain size does also not change this result – there is no difference in the slope of the relationship between brain size and thumb length for tool-using primates.

In short, there is no difference in thumb length among species that use tools – at least not among extant primates. The debate on which extinct apes and hominins are likely to have been capable of human-like precision grasping behaviours is extensive and ongoing [16,27,34-36]. Our results highlight that, in isolation, no single morphological feature – including thumb length – is likely to be informative as to the tool-making behaviour of individual species [26]. Instead, it is more likely that a suite of complex characteristics and morphologies [26,29] have given rise to a variety of manipulative abilities associated with extractive foraging – only some of which require tool use.

Supplementary Note 3: Peak workspace

Some authors argue that having a relatively long thumb does not necessarily lead to high manipulation ability and that we should instead quantify manipulation ability using kinematic models[16]. In the kinematic model proposed by Feix et al.[16], a precision-grip *workspace* is calculated across a circular object of varying size scaled to hand size, resulting in a range of workspace values relative to a given object size. A '*manipulation workspace'* can be defined as the range of motion a small object can be freely moved between the thumb and index finger[16]. One way of summarizing this is to look at the 'peak workspace', i.e. the object size at which a species (or specimen) has the highest workspace value.

We sought to demonstrate a significant association between brain size and peak workspace (accounting for object size) using a reduced sample of 41 primates with workspace data[16] – and accounting for the optimum object size which varies among species. To do this, we ran phylogenetic regression models in the same way as those we present in the main text. In these models, our response variable is the peak precision workspace, and our predictor variables are brain size and object size. All variables are $\log_{10}$ transformed.

We find that brain size is a significant predictor of peak workspace ($\beta_{[brain]}$ = 0.09, p_x<0.05 in 100% of topologies). The result is qualitatively identical when *H. sapiens* are excluded ($\beta_{[brain]}$ =0.09-

0.11, p_x<0.05 in 100% of topologies). We also find a significant negative effect of object size on workspace ($\beta_{[object\ size]}$ between -0.15 and -0.14, p_x<0.05 in 100% of topologies).

Given these relationships, we therefore can conclude that peak workspace and relative thumb length are both reasonable proxies for overall manual dexterity across primates. Furthermore, there is a strong and significant association between the thumb length and peak workspace of each species. Including workspace as a covariate into our finger-only models demonstrates a highly significant association between the two variables ($\beta_{[workspace]}$=0.57-0.61, p_x<0.05 in 100% of topologies) that is unaffected by the inclusion of *H. sapiens* or object size.

Whilst kinematic models are an elegant mechanical way to reconstruct and think about manipulative ability in individuals, they can be quite difficult to interpret or summarise into quantities that fit the expectations of evolutionary models. For example, modern *H. sapiens* have greater estimated manipulation potential than Neanderthals[16] – consistent with the idea that Neanderthals were more adapted for power gripping[37,38] though see[39]. However, the 'optimal' object size for *H. sapiens* is 19mm compared to the Neanderthal's tiny 3mm. Biologically speaking, it is very unlikely that the hands of Neanderthals were under strong selection to specifically manipulate objects of 3mm in size.

Supplementary Note 4: Brain regions and binocularity

We might expect that any improvements in fine-grained visuo-motor processes such as manual dexterity may have been driven by correlated increases in the regions of the brain responsible for both visual (e.g., the neocortex) and motor (e.g., the cerebellum) control.

The brain region data was taken from published literature[40-42] and consists of neocortex volume and cerebellum volume for a total of 49 species for which we have metacarpal data and whole brain sizes (see **Supplementary Dataset 1**). These are all extant taxa – we have no data for any hominins except modern *H. sapiens*.

In a subset of the whole-brain data for which we have data on the volume of both the neocortex and cerebellum (n = 49), we identified a significant positive relationship between both finger length ($\beta_{[finger]}$ = 0.735, p_x < 0.05 in 100% of trees) and neocortex ($\beta_{[neocortex]}$ = 0.16-0.20, p_x < 0.05 in 100% of trees, **Figure 4**).

Surprisingly, there is no significant association found with the cerebellum in any model on any tree. Although correlated evolution of neocortex and cerebellum is a pronounced feature of primate brain evolution[43,44], *H. sapiens* and other apes deviate from this pattern, exhibiting relative expansion of the cerebellum[40]. However, there is no significant association between relative thumb length and cerebellum size regardless of the inclusion or exclusion of apes (and the sample size is too small to test in isolation, n = 6). The parameters of these models are presented in **Supplementary Table 1**.

Separating the effects of the neocortex and the cerebellum can be complicated owing to their strong correlation. Here, we include both regions in the same model since together, the neocortex and cerebellum comprise a 'unit' responsible for the mediation of visuo-motor and sequential action control[45]. However, we find qualitatively similar results when each of the regions are studied in isolation: without *H. sapiens*, only the neocortex shows any significant association (**Supplementary Table 8**).

Supplementary Table 8

Parameter estimates for results using only a single brain region as predictor variables.

	R^2	λ	β	p_x	β	p_x	β	p_x
			Finger		Neocortex		Cerebellum	
1	0.91-0.92	0.94-0.98	0.70-0.73	0 [100]	0.12-0.14	0 [100]	-	-
2	0.96-0.96	0.97-0.98	0.80-0.84	0 [100]	0.05-0.07	0.03-0.07 [95]	-	-
3	0.90-0.92	0.91-0.97	0.73-0.77	0 [100]	-	-	0.10-0.12	0.00-0.01 [100]
4	0.96-0.97	0.96-0.98	0.85-0.89	0 [100]	-	-	0.03-0.05	0.08-0.14 [0]

Note. All values reported as the range of median values observed across the posterior distribution estimated for each of 100 trees. For p_x values, we also report the number of trees in which less than 5% of the posterior distribution crosses zero in square brackets. Models are as follows: 1, neocortex only (N = 49); 2, neocortex only excluding *H. sapiens* (N = 48); 3, cerebellum only (N = 49); 4, cerebellum only excluding *H. sapiens* (N = 48). In all models, finger length is still included as a covariate.

Manipulation is visually guided. As brain size and binocularity has been linked through the evolutionary expansion of visual regions[46,47], we additionally tested for an association with binocular field overlap and thumb length. Taking data from the literature on the degree of orbital convergence[48], we have 39 species that overlap with our thumb length and brain size data (see **Supporting Dataset 1**). Note that this does not include any extinct taxa, nor is there data for *Homo sapiens*. We recover the expected association between brain size and binocularity[46]: Both body size and binocularity are significant predictors of whole brain size in 100% of topologies (β_{body} =0.55-0.56, β_{binoc} = 0.79-1.02). In terms of thumb length, the relationship with brain size is retained in this reduced sample, but there is no significant relationship with binocularity – either when studied in isolation or with brain size. In short, binocularity is associated with brain size, and so is thumb length – but they are not associated with one another.

We might expect that differences observed amongst primates might lead to non-significance in the cerebellum across primates. Strepsirrhines have relatively small neocortices in comparison to other primates[49]. However, the results are qualitatively identical when we study the above relationships in strepsirrhines and haplorrhines separately.

All our results point to a singular conclusion: that neocortex size is implicated in thumb length evolution across primates, but not cerebellum size. However, further attention is required to untangle the specific effects of cortical regions on thumb length across primates.

Supplementary References

1 Wisniewski, A. L., Lloyd, G. T. & Slater, G. J. Extant species fail to estimate ancestral geographical ranges at older nodes in primate phylogeny. *Proceedings of the Royal Society B: Biological Sciences* **289**, 20212535 (2022). https://doi.org/10.1098/rspb.2021.2535

2 Boyer, D. M., Yapuncich, G. S., Chester, S. G. B., Bloch, J. I. & Godinot, M. in *The Evolution of the Primate Hand Developments in Primatology: Progress and Prospects* Ch. Chapter 14, 373-419 (2016).

3 Boyer, D. M., Seiffert, E. R., Gladman, J. T. & Bloch, J. I. Evolution and allometry of calcaneal elongation in living and extinct primates. *PLoS ONE* **8** (2013). https://doi.org/10.1371/journal.pone.0067792

4 Hart, E. N. *Metacarpal ratio and its relation to sexual dimorphism in primates with different mating strategies*. (Louisiana State University and Agricultural & Mechanical College, 2018).

5 Kivell, T. L. *et al.* The hand of *Homo naledi*. *Nature communications* **6** (2015). https://doi.org/10.1038/ncomms9431

6 Prang, T. C., Ramirez, K., Grabowski, M. & Williams, S. A. Ardipithecus hand provides evidence that humans and chimpanzees evolved from an ancestor with suspensory adaptations. *Sci Adv* **7** (2021). https://doi.org/10.1126/sciadv.abf2474

7 Lemelin, P. *The evolution of manual prehensility in primates: a comparative study of prosimians and didelphid marsupials*. (State University of New York at Stony Brook, 1996).

8 Bouckaert, R. *et al.* BEAST 2: a software platform for Bayesian evolutionary analysis. *PLoS Computational Biology* **10**, e1003537 (2014). https://doi.org/10.1371/journal.pcbi.1003537

9 Avaria-Llautureo, J. *et al.* The radiation and geographic expansion of primates through diverse climates. *Proceedings of the National Academy of Sciences* **122**, e2423833122 (2025). https://doi.org/doi:10.1073/pnas.2423833122

10 Heath, T. A., Huelsenbeck, J. P. & Stadler, T. The fossilized birth–death process for coherent calibration of divergence-time estimates. *Proceedings of the National Academy of Sciences USA* **111**, 2957-2966 (2014). https://doi.org/10.1073/pnas.1319091111

11 Dos Reis, M. *et al.* Using phylogenomic data to explore the effects of relaxed clocks and calibration strategies on divergence time estimation: Primates as a test case. *Systematic Biology* **67**, 594-615 (2018). https://doi.org/10.1093/sysbio/syy001

12 Kendall, M. & Colijn, C. Mapping phylogenetic trees to reveal distinct patterns of evolution. *Molecular biology and evolution* **33**, 2735-2743 (2016).

13 Jombart, T., Kendall, M., Almagro-Garcia, J. & Colijn, C. treespace: Statistical exploration of landscapes of phylogenetic trees. *Molecular ecology resources* **17**, 1385-1392 (2017).

14 R: A language and environment for statistical computing (R Foundation for Statistical Computing, 2024).

15 Almécija, S., Wallace, I. J., Judex, S., Alba, D. M. & Moyà-Solà, S. Comment on "Human-like hand use in *Australopithecus africanus*". *Science* **348**, 1101 (2015). https://doi.org/10.1126/science.aaa8414

16 Feix, T., Kivell, T. L., Pouydebat, E. & Dollar, A. M. Estimating thumb–index finger precision grip and manipulation potential in extant and fossil primates. *Journal of the Royal Society Interface* **12** (2015). https://doi.org/10.1098/rsif.2015.0176
17 Melin, A. D. *et al.* Anatomy and dietary specialization influence sensory behaviour among sympatric primates. *Proceedings of the Royal Society B: Biological Sciences* **289**, 20220847 (2022). https://doi.org/doi:10.1098/rspb.2022.0847
18 Kjaer, M. S. & Kjaer, I. Human fetal hand size and hand maturity in the first half of the prenatal period. *Early Human Development* **50**, 193-207 (1998). https://doi.org/https://doi.org/10.1016/S0378-3732(97)00039-8
19 Zaher, J. F., El-Ameen, N. F. M. & Seedhom, A. E. Stature estimation using anthropometric measurements from computed tomography of metacarpal bones among Egyptian population. *Egyptian Journal of Forensic Sciences* **1**, 103-108 (2011). https://doi.org/https://doi.org/10.1016/j.ejfs.2011.03.002
20 Orland, M. D. *et al.* Hand size affects branching of the deep ulnar nerve and deep palmar arch. *Surgical and Radiologic Anatomy* **44**, 1501-1505 (2022). https://doi.org/10.1007/s00276-022-03043-1
21 Alba, D. M., Moyà-Solà, S. & Köhler, M. Morphological affinities of the *Australopithecus afarensis* hand on the basis of manual proportions and relative thumb length. *Journal of Human Evolution* **44**, 225-254 (2003). https://doi.org/10.1016/S0047-2484(02)00207-5
22 Green, D. J. & Gordon, A. D. Metacarpal proportions in *Australopithecus africanus*. *Journal of Human Evolution* **54**, 705-719 (2008). https://doi.org/https://doi.org/10.1016/j.jhevol.2007.10.007
23 Pouydebat, E., Laurin, M., Gorce, P. & Bels, V. Evolution of grasping among anthropoids. *Journal of Evolutionary Biology* **21**, 1732-1743 (2008). https://doi.org/https://doi.org/10.1111/j.1420-9101.2008.01582.x
24 caper: Comparative Analyses of Phylogenetics and Evolution in R. v. R package version 0.5. (2012).
25 Tague, R. G. Variability of a vestigial structure: first metacarpal in *Colobus guereza* and *Ateles geoffroyi*. *Evolution* **51**, 595-605 (1997). https://doi.org/10.1111/j.1558-5646.1997.tb02446.x
26 Marzke, M. W. Precision grips, hand morphology, and tools. *American Journal of Physical Anthropology* **102**, 91-110 (1997). https://doi.org/10.1002/(SICI)1096-8644(199701)102:1<91::AID-AJPA8>3.0.CO;2-G
27 Almécija, S., Moyà-Solà, S. & Alba, D. M. Early origin for human-like precision grasping: A comparative study of pollical distal phalanges in fossil hominins. *PLoS ONE* **5**, e11727 (2010). https://doi.org/10.1371/journal.pone.0011727
28 Susman, R. L. Fossil evidence for early hominid tool use. *Science* **265**, 1570-1573 (1994). https://doi.org/doi:10.1126/science.8079169
29 Marzke, M. W. Tool making, hand morphology and fossil hominins. *Philosophical Transactions of the Royal Society B: Biological Sciences* **368** (2013). https://doi.org/10.1098/rstb.2012.0414
30 Shumaker, R. W., Walkup, K. R. & Beck, B. B. *Animal tool behavior: the use and manufacture of tools by animals*. (JHU Press, 2011).
31 St Amant, R. & Horton, T. E. Revisiting the definition of animal tool use. *Animal Behaviour* **75**, 1199-1208 (2008). https://doi.org/https://doi.org/10.1016/j.anbehav.2007.09.028
32 Beck, B. B. Animal tool behavior: The use and manufacture of tools by animals. *(No Title)* (1980).
33 Bentley-Condit, V. Animal tool use: current definitions and an updated comprehensive catalog. *Behaviour* **147**, 185-132A (2010).

34 Moyà-Solà, S., Köhler, M. & Rook, L. Evidence of hominid-like precision grip capability in the hand of the Miocene ape *Oreopithecus*. *Proceedings of the National Academy of Sciences USA* **96**, 313-317 (1999). https://doi.org/10.1073/pnas.96.1.313
35 Almécija, S. & Alba, D. M. On manual proportions and pad-to-pad precision grasping in Australopithecus afarensis. *J. Hum. Evol* **73**, 88-92 (2014).
36 Skinner, M. M. *et al.* Human-like hand use in *Australopithecus africanus*. *Science* **347**, 395-399 (2015). https://doi.org/10.1126/science.1261735
37 Niewoehner, W. Neanderthal hands in their proper perspective. *Neanderthals revisited: New approaches and perspectives*, 157-190 (2006).
38 Bardo, A. *et al.* The implications of thumb movements for Neanderthal and modern human manipulation. *Scientific reports* **10**, 19323 (2020).
39 Karakostis, F. A., Hotz, G., Tourloukis, V. & Harvati, K. Evidence for precision grasping in Neandertal daily activities. *Science Advances* **4**, eaat2369 (2018).
40 Barton, R. A. & Venditti, C. Rapid evolution of the cerebellum in humans and other great apes. *Current Biology* **24**, 2440-2444 (2014). https://doi.org/10.1016/j.cub.2014.08.056
41 Frahm, H., Stephan, H. & Stephan, M. Comparison of brain structure volumes in Insectivora and Primates. I. Neocortex. *Journal fur Hirnforschung* **23**, 375-389 (1982).
42 Stephan, H., Frahm, H. & Baron, G. New and revised data on volumes of brain structures in insectivores and primates. *Folia primatologica* **35**, 1-29 (1981).
43 Barton, R. A. Embodied cognitive evolution and the cerebellum. *Philosophical Transactions of the Royal Society B: Biological Sciences* **367**, 2097-2107 (2012).
44 Whiting, B. & Barton, R. The evolution of the cortico-cerebellar complex in primates: anatomical connections predict patterns of correlated evolution. *Journal of human evolution* **44**, 3-10 (2003).
45 Wagner, M. J. & Luo, L. Neocortex–cerebellum circuits for cognitive processing. *Trends in neurosciences* **43**, 42-54 (2020).
46 Barton, R. A. Binocularity and brain evolution in primates. *Proceedings of the National Academy of Sciences USA* **101**, 10113-10115 (2004). https://doi.org/10.1073/pnas.0401955101
47 Barton, R. A. Visual specialization and brain evolution in primates. *Proceedings of the Royal Society of London. Series B: Biological Sciences* **265**, 1933-1937 (1998).
48 Ross, C. F. Allometric and functional influences on primate orbit orientation and the origins of the Anthropoidea. *Journal of Human Evolution* **29**, 201-227 (1995).
49 DeCasien, A. R. & Higham, J. P. Primate mosaic brain evolution reflects selection on sensory and cognitive specialization. *Nature Ecology & Evolution* **3**, 1483-1493 (2019).
