# Supplementary material for: Human dexterity and brains evolved hand in hand
Source: Commun Biol. 2025 Aug 26;8:1257. doi: 10.1038/s42003-025-08686-5 (PMC12381199; doi:10.1038/s42003-025-08686-5)
Supplement: Supplementary file 2 — Description of Additional Supplementary Files [file 42003_2025_8686_MOESM2_ESM.pdf]

Description of Additional Supplementary Files

File name: Supplementary Data 1

Description: The full dataset analyzed

File name: Supplementary Data 2

Description: The sample of trees used
