# Supplementary material for: Human dexterity and brains evolved hand in hand
Source: Commun Biol. 2025 Aug 26;8:1257. doi: 10.1038/s42003-025-08686-5 (PMC12381199; doi:10.1038/s42003-025-08686-5)
Supplement: Supplementary file 5 — Reporting Summary [file 42003_2025_8686_MOESM5_ESM.pdf]

nature portfolio

Corresponding author(s): Joanna Baker and Chris Venditti

Last updated by author(s): 17/06/2025

Reporting Summary

Nature Portfolio wishes to improve the reproducibility of the work that we publish. This form provides structure for consistency and transparency in reporting. For further information on Nature Portfolio policies, see our Editorial Policies and the Editorial Policy Checklist.

Statistics

For all statistical analyses, confirm that the following items are present in the figure legend, table legend, main text, or Methods section.

n/a	Confirmed	
☐	☒	The exact sample size (*n*) for each experimental group/condition, given as a discrete number and unit of measurement
☐	☒	A statement on whether measurements were taken from distinct samples or whether the same sample was measured repeatedly
☐	☒	The statistical test(s) used AND whether they are one- or two-sided<br>*Only common tests should be described solely by name; describe more complex techniques in the Methods section.*
☐	☒	A description of all covariates tested
☐	☒	A description of any assumptions or corrections, such as tests of normality and adjustment for multiple comparisons
☐	☒	A full description of the statistical parameters including central tendency (e.g. means) or other basic estimates (e.g. regression coefficient) AND variation (e.g. standard deviation) or associated estimates of uncertainty (e.g. confidence intervals)
☐	☒	For null hypothesis testing, the test statistic (e.g. *F*, *t*, *r*) with confidence intervals, effect sizes, degrees of freedom and *P* value noted<br>*Give P values as exact values whenever suitable.*
☐	☒	For Bayesian analysis, information on the choice of priors and Markov chain Monte Carlo settings
☒	☐	For hierarchical and complex designs, identification of the appropriate level for tests and full reporting of outcomes
☒	☐	Estimates of effect sizes (e.g. Cohen's *d*, Pearson's *r*), indicating how they were calculated

Our web collection on statistics for biologists contains articles on many of the points above.

Software and code

Policy information about availability of computer code

Data collection	No software was used for data collection.
Data analysis	All analyses in this study were performed using openly accessible software as cited in the main text.

For manuscripts utilizing custom algorithms or software that are central to the research but not yet described in published literature, software must be made available to editors and reviewers. We strongly encourage code deposition in a community repository (e.g. GitHub). See the Nature Portfolio guidelines for submitting code & software for further information.

Data

Policy information about availability of data

All manuscripts must include a data availability statement. This statement should provide the following information, where applicable:

- Accession codes, unique identifiers, or web links for publicly available datasets
- A description of any restrictions on data availability
- For clinical datasets or third party data, please ensure that the statement adheres to our policy

The authors declare that data supporting the finding of this study is available in public databases and in previous studies listed in the references. The full dataset is provided as supporting information.

April 2023

Research involving human participants, their data, or biological material

Policy information about studies with human participants or human data. See also policy information about sex, gender (identity/presentation), and sexual orientation and race, ethnicity and racism.

Reporting on sex and gender	Does not apply for our study.
Reporting on race, ethnicity, or other socially relevant groupings	Does not apply for our study.
Population characteristics	Does not apply for our study.
Recruitment	Does not apply for our study.
Ethics oversight	Does not apply for our study.

Note that full information on the approval of the study protocol must also be provided in the manuscript.

Field-specific reporting

Please select the one below that is the best fit for your research. If you are not sure, read the appropriate sections before making your selection.

☐ Life sciences ☐ Behavioural & social sciences ☒ Ecological, evolutionary & environmental sciences

For a reference copy of the document with all sections, see nature.com/documents/nr-reporting-summary-flat.pdf

Ecological, evolutionary & environmental sciences study design

All studies must disclose on these points even when the disclosure is negative.

Study description	We used Bayesian phylogenetic comparative methods to study the relationship between thumb length and brain size in primates.
Research sample	A total of 95 extant and extinct species with brain size and digit length data.
Sampling strategy	We used all available data from previously published literature. Species-level averages were used.
Data collection	Data was was collected and checked by JB and CV.
Timing and spatial scale	Data was collected from public data bases and published literature. Details are available in the Methods section.
Data exclusions	We excluded Colobus monkeys owing to their malformed thumbs although analyses were identical with them included. We also excluded spider monkeys as they did not have thumb metacarpals.
Reproducibility	All analyses were conducted multiple times to ensure repeatability.
Randomization	This is not relevant to our study because it is not experimental in this way.
Blinding	This is not relevant to our study because it is not experimental in this way.

Did the study involve field work? ☐ Yes ☒ No

Reporting for specific materials, systems and methods

We require information from authors about some types of materials, experimental systems and methods used in many studies. Here, indicate whether each material, system or method listed is relevant to your study. If you are not sure if a list item applies to your research, read the appropriate section before selecting a response.

Materials & experimental systems

n/a	Involved in the study
☒	☐ Antibodies
☒	☐ Eukaryotic cell lines
☒	☐ Palaeontology and archaeology
☒	☐ Animals and other organisms
☒	☐ Clinical data
☒	☐ Dual use research of concern
☒	☐ Plants

Methods

n/a	Involved in the study
☒	☐ ChIP-seq
☒	☐ Flow cytometry
☒	☐ MRI-based neuroimaging

Plants

Seed stocks	Does not apply for our study.
Novel plant genotypes	Does not apply for our study.
Authentication	Does not apply for our study.
